# The Synthesis, Characterization, and Theoretical Study of Ruthenium (II) Polypyridyl Oligomer Hybrid Structures with Reduced Graphene Oxide for Enhanced Optoelectronic Applications

**DOI:** 10.3390/ijms252312989

**Published:** 2024-12-03

**Authors:** Alexander Schultheiss, Jamel White, Khoa Le, Nicole Boone, Ufana Riaz, Darlene K. Taylor

**Affiliations:** Department of Chemistry and Biochemistry, North Carolina Central University, Durham, NC 27707, USA

**Keywords:** conjugated polymers, ruthenium, bipyridyl, semiconductor, metal transfer, organic chromophores, Suzuki coupling, energy storage

## Abstract

π-conjugated polymers are arguably one of the most exciting classes of materials and have attracted substantial attention due to their unique optical and electronic properties. The introduction of transition metals into conjugated polymers tunes the optoelectronic properties of these metallopolymers, which may improve their performance in device applications. Graphene and reduced graphene oxide (RGO) derivatives are interesting materials with a unique structure and outstanding properties. The present work reports an investigation of three hybrid RGO and π-conjugated oligomers that contain ruthenium polypyridyl chromophores serving as models to provide molecular-level insight for the corresponding transition-metal-containing conjugated polymers.

## 1. Introduction

As the global population continues to grow at an unprecedented rate, the demand for energy is rapidly increasing. This rising energy demand places significant pressure on nonrenewable resources, particularly fossil fuels, which are both limited and environmentally harmful [1,2]. To meet this escalating need, it is essential to explore and develop renewable energy sources that are not only efficient but also sustainable and environmentally friendly. Among these, harnessing solar energy to generate clean, renewable electricity presents a promising pathway toward reducing our reliance on fossil fuels and mitigating the environmental impacts of energy production [3,4,5]. To achieve this vision, researchers are actively seeking a way to replicate the natural process of photosynthesis, in which sunlight is absorbed by chlorophyll molecules to drive the conversion of light energy into chemical energy. In photosynthesis, sunlight excites electrons in chlorophyll, which are then transferred to electron-deficient molecules, with electrons from water molecules filling the void left behind, initiating a series of chemical reactions that ultimately produce energy [6,7]. By mimicking this process, it may be possible to develop artificial systems for efficient solar energy conversion.

In our previous work with para-phenylene dimers, we demonstrated that the electronic properties of para-phenylene systems can be tuned by introducing specific substitutions and coupling them with π-acceptors [8]. We found that the band gap of an unsubstituted para-phenylene (4.13 eV) could be reduced to 2.9 eV when linked to a diphenylamine-substituted para-phenylene and a π-acceptor. Furthermore, we observed significant fluorescence quenching when the para-phenylene dimer was mixed with reduced graphene oxide (RGO), suggesting a phenomenon of excited-state electron transfer. This finding prompted us to explore the effects of RGO on polypyridyl Ru(II)-based systems, specifically aiming to understand and control the interactions between these materials. To facilitate efficient charge transport and improve system mobility, we designed our Ru(II) complex with a conjugated backbone, which enhances π–π interactions. The primary goal of this study was to investigate the electron and energy transfer dynamics in novel Ru(II) polypyridyl oligomers, particularly in the presence of reduced graphene oxide (RGO). Polypyridyl ligands, particularly those with electron-donating groups like -NH_2_ or -OCH_3_, can donate electron density to the metal center via their nitrogen lone pairs. This can alter the oxidation state of the ruthenium center, often stabilizing a lower oxidation state (such as Ru^2+^) and influencing the electronic structure of the complex. These ligands can absorb light, and their electronic structure allows them to participate in ligand-to-metal charge transfer (LMCT) transitions, where an electron is excited from the ligand (usually the nitrogen lone pair of the pyridine) to the metal center. In ruthenium complexes, these LMCT transitions can often occur in the visible-to-UV region and can significantly influence optical properties, contributing to the color and light absorption characteristics of the complex. Conversely, the ruthenium metal center can also participate in metal-to-ligand charge transfer (MLCT) transitions, where electrons are excited from the metal’s d-orbitals to the π* orbitals of the ligands. The presence of polypyridyl ligands enhances the possibility of these MLCT transitions, which are typically responsible for strong visible absorption bands in ruthenium-based complexes. Ruthenium polypyridyl complexes exhibit luminescence properties, often with long-lived excited states due to the strong spin–orbit coupling of the ruthenium metal center. The ligands can also modulate the emission wavelength and quantum yield by influencing the metal’s electronic structure and the rates of non-radiative decay. This is important for applications such as catalysis, electrochemical sensing, or energy storage. Therefore, the redox potentials of ruthenium-based complexes can be tuned by modifying the ligand structure. These interactions are crucial for the development of more-efficient energy conversion systems, such as light-driven catalytic water oxidation in photovoltaic cells. The nature of the interactions between the oligomers and RGO can vary from covalent bonding to electrostatic interactions and can occur over distances ranging from a short range to several nanometers [9,10,11,12]. Understanding these interactions will provide insights that can guide the design of more-effective devices for solar energy conversion. The study aims to evaluate the potential of graphene and graphene-based hybrid materials as electron-accepting components in energy systems. Given graphene’s excellent electrical conductivity, mechanical strength, and availability, it is an ideal material for creating efficient hybrid composites. These materials are expected to improve the solubility and processability of graphene, making it a versatile component in various applications, including energy storage and solar energy harvesting [13,14,15]. In this context, we developed two graphene-hybrid composites to study electron/energy transfer interactions. Specifically, we modified Hummers’ and Offeman’s method to oxidize graphite and employed a reducing agent to obtain RGO. We propose that RGO can serve as an effective alternative to TiO_2_ in future artificial light-harvesting systems, enhancing their efficiency and performance.

## 2. Results

### 2.1. Reaction Schemes for A1[Ru(bpy)_2_]^2+^ and A2[Ru(bpy)_2_]^2+^

**Scheme 1:**
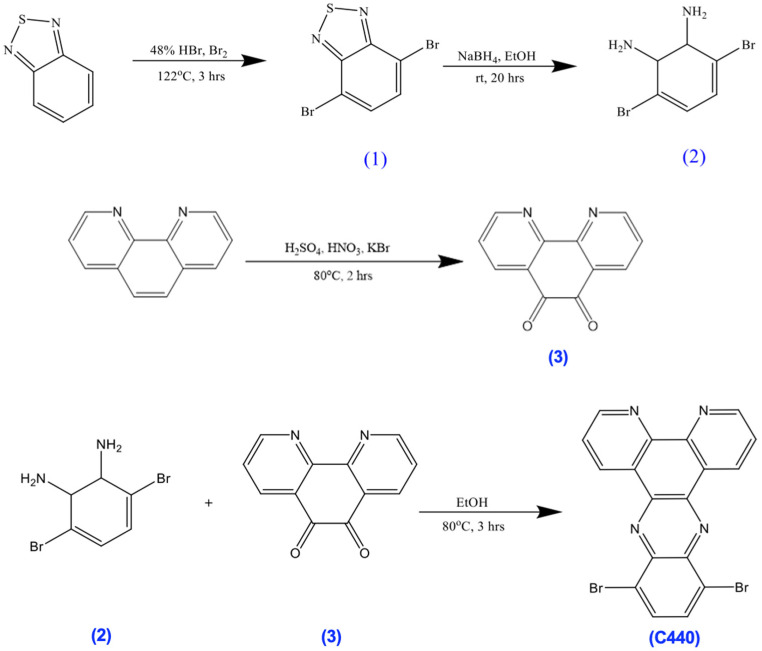

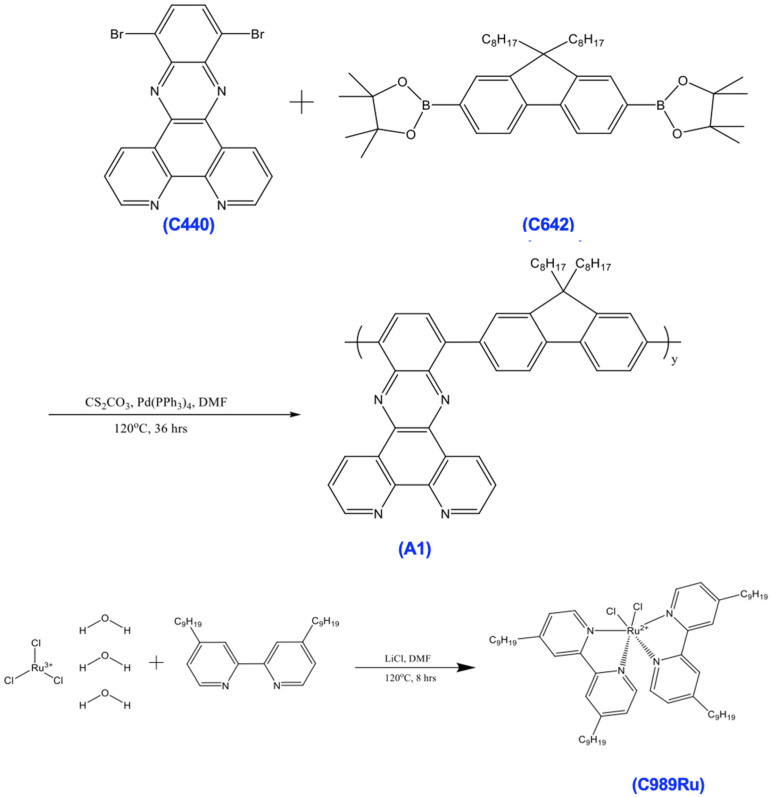

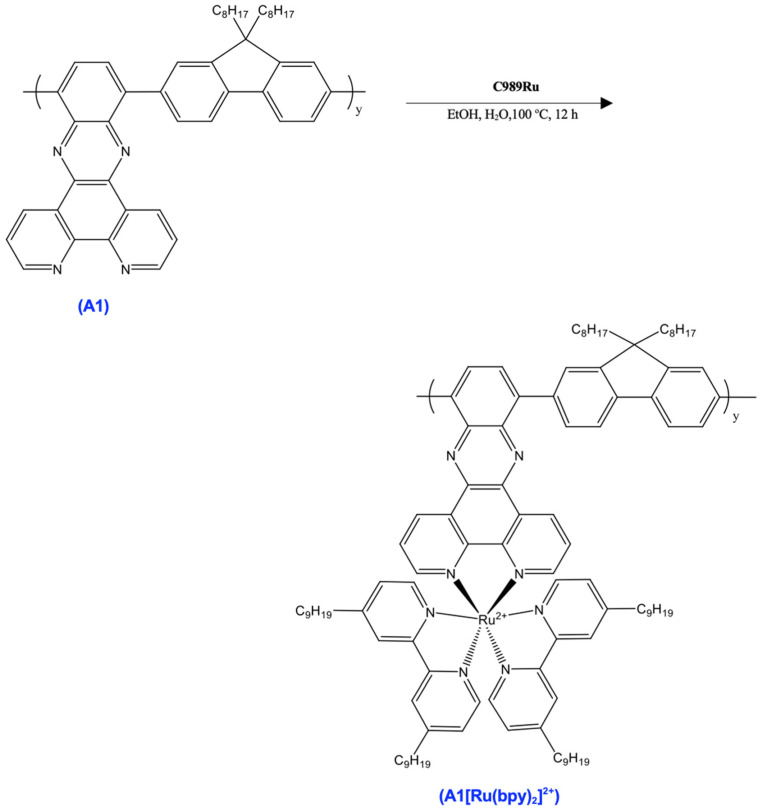
Reaction scheme for A1[Ru(bpy)_2_]^2+^.

**Scheme 2:**
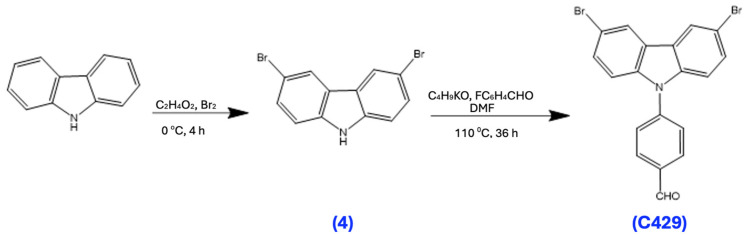

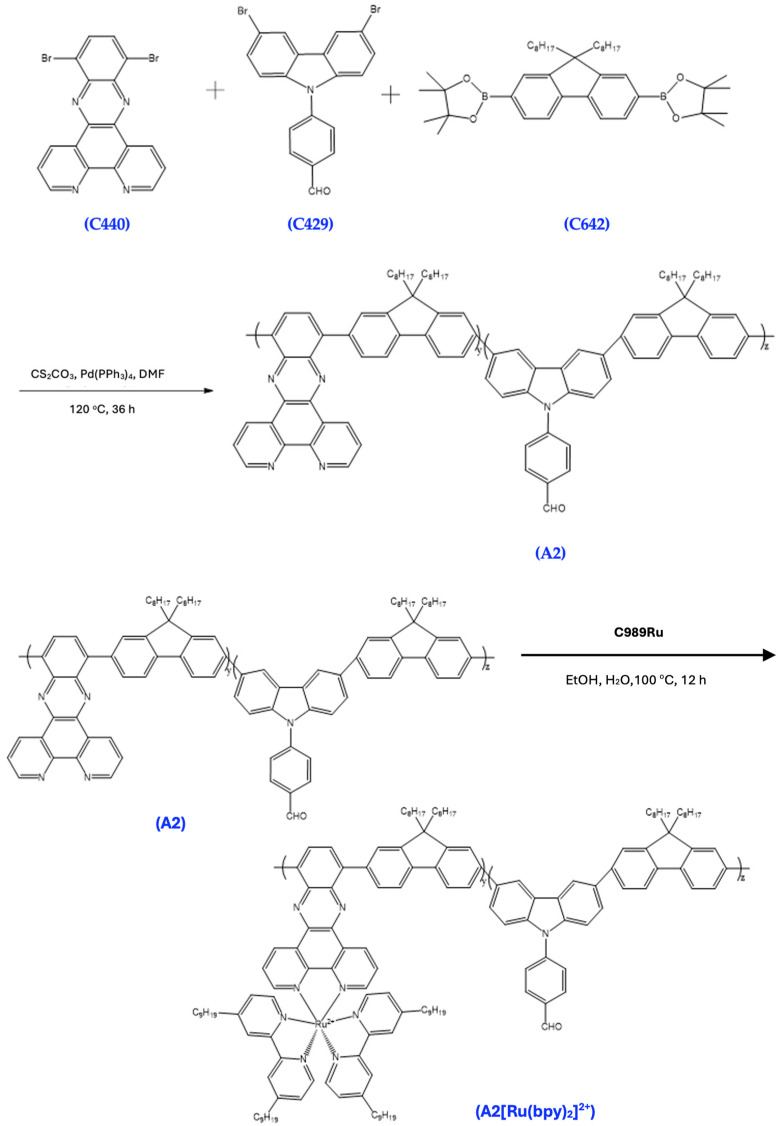
Reaction scheme for A2[Ru(bpy)_2_]^2+^.

The product A1[Ru(bpy)_2_]^2+^ was synthesized in six steps (see Scheme 1). A white needle-like precipitate was obtained in good yield (88%) by brominating commercially available 1,2,5-benzothiadiazole. Due to its low solubility, recrystallization of the compound (compound **1**) required methanol (MeOH), which was chosen because it effectively dissolved the impurities in the crude product. The reduction of compound 1 using sodium borohydride (NaBH_4_) produced a white solid, compound **2**, identified as 1,2-diamine. In the reaction flask containing compound **2**, a 16-gauge needle was inserted into the septum to allow gas release during the reaction. Compound **3** was synthesized with high yield (95%) by oxidizing 1,10-phenanthroline with KBr in a mixture of aqueous sulfuric and nitric acids, followed by refluxing to yield a yellow product. A condensation reaction between compounds **2** and **3** resulted in the formation of a yellow quinoxaline-based spacer, C440. A Suzuki coupling reaction between the monomer C440 and commercially available C642 in the presence of the catalyst tetrakis(triphenylphosphine)palladium(0) yielded an orange/brown solid A1. A1 is an alternating co-block oligomer formed because the di-halogenated C440 can only bind to an available boric acid site on C642, and vice versa. The choice to use C642, a co-monomer with long alkyl units, was intended to improve the solubility of the final oligomer. A1 was purified by Soxhlet extraction for 3 days, leaving an orange solid in the thimble. C989Ru was prepared following a standard procedure from the literature [14]. The desired final product, A1[Ru(bpy)_2_]^2+^, was obtained by refluxing a mixture of C989Ru and A1 in ethanol under dry nitrogen. The inclusion of long alkyl units in C989Ru facilitated the solubilization of A1, which otherwise had poor solubility. Scheme 2 highlights the reactions involved in the formation of A2[Ru(bpy)_2_]^2+^. Compound 4 was prepared with good yield (79%) by brominating commercially available carbazole, resulting in a white solid. The addition of 4-fluorobenzaldehyde to the secondary amine on compound 4 led to the formation of the desired product C429. Oligomer A2 was synthesized through a Suzuki coupling reaction between monomers C440, C429, and C642, with tetrakis(triphenylphosphine)palladium(0) as the catalyst (Scheme 2). The resulting A2 is a randomized block oligomer, formed due to competing dibromo-substituted monomers (C440 and C429) attaching to limited boric acid sites on C642.

**Scheme 3:**
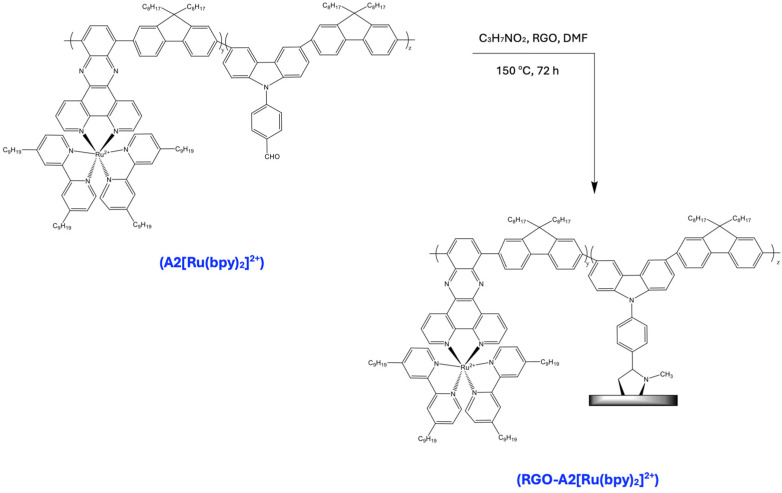
Reaction scheme for grafting A2[Ru(bpy)_2_]^2+^ to RGO.

The end capping of A2 was achieved by refluxing the mixture of A2 and C989Ru in ethanol to yield A2[Ru(bpy)_2_]^2+^ (see Scheme 3). The final product, RGO-A2[Ru(bpy)_2_]^2+^, was obtained through a 1,3-dipolar cycloaddition reaction between sarcosine, reduced graphene oxide (RGO), and A2[Ru(bpy)_2_]^2+^. The crude RGO-A2[Ru(bpy)_2_]^2+^ was thoroughly washed with ethanol and dichloromethane (DCM) to remove any unattached A2[Ru(bpy)_2_]^2+^.

### 2.2. UV–Visible Studies

**Figure 1:**
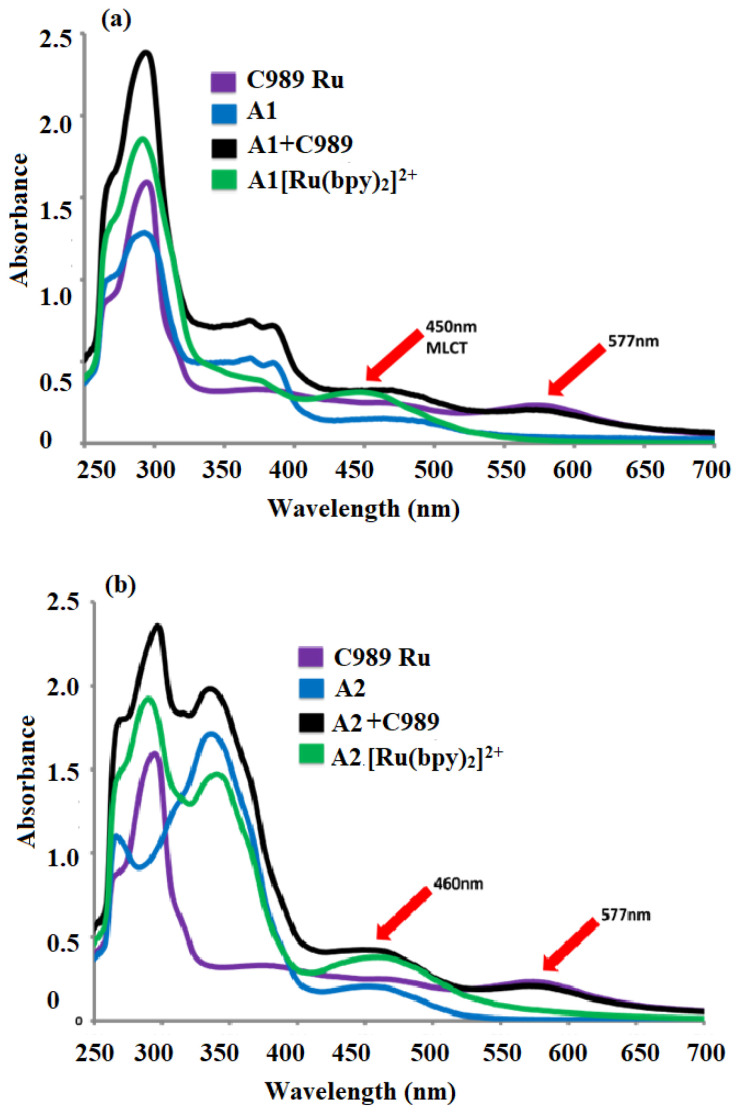
UV–visible spectra of (**a**) C989Ru, A1, a mixture of A1 + C989Ru, and A1[Ru(bpy)_2_]^2+^; (**b**) C989Ru, A2, a mixture of A^2+^ C989Ru, and A2[Ru(bpy)_2_]^2+^. Note the red arrows draw attention to the peaks of interest.

The absorbance spectra of C989Ru, A1, a mixture of A1 + C989Ru, and A1[Ru(bpy)_2_]^2+^ were measured in DMF using a quartz sample cell with a 1 cm path length. The results are shown in Figure 1a. The assignment of the absorption bands for the complex A1[Ru(bpy)_2_]^2+^ was based on the well-established optical transitions observed in analogous Ru(II) polypyridyl complexes [15,16,17]. The 290 nm band in the spectra of C989Ru, A1 + C989Ru, and A1[Ru(bpy)_2_]^2+^ is attributed to intra-ligand π–π* transitions within the 2,2′-bipyridine ligand [17]. The 375 nm band observed in the spectra of A1, A1 + C989Ru, and A1[Ru(bpy)_2_]^2+^ is assigned to transitions involving the phenanthroline ligand incorporated into the oligomer backbone. In the spectra of A1[Ru(bpy)_2_]^2+^ and A1 + C989Ru, the 450 nm band corresponds to a metal-to-ligand charge transfer (MLCT) transition, specifically the dπ(Ru) → π* transition [17,18]. However, in the spectrum of C989Ru, this transition is redshifted to 577 nm. The presence of a 450 nm band in A1 (which does not contain ruthenium) suggests that an unidentified component in the oligomer backbone contributes to this absorption. The mixture of A1 + C989Ru shows both the 450 nm and 577 nm bands, indicating that part of the C989Ru is interacting with A1, leading to the 450 nm transition, while some C989Ru remains unbound, contributing to the 577 nm absorption. The absence of the 577 nm band in the spectrum of A1[Ru(bpy)_2_]^2+^ suggests that all C989Ru in the final complex is fully coordinated with A1. Similarly, the absorbance spectra of C989Ru, A2, a mixture of A2 + C989Ru, and A2[Ru(bpy)_2_]^2+^ were obtained in DMF and recorded using a quartz cell with a 1 cm path length on a Molecular Devices SpectraMax M5 UV-Vis spectrophotometer, as shown in Figure 1b. As with A1, the absorption band assignments for complex A2[Ru(bpy)_2_]^2+^ were made based on similar transitions seen in Ru(II) polypyridyl complexes. The 290 nm band in the spectra of C989Ru, A2 + C989Ru, and A2[Ru(bpy)_2_]^2+^ is attributed to intraligand π–π* transitions in the 2,2′-bipyridine ligand [17]. The 335 nm band in the A2, A2 + C989Ru, and A2[Ru(bpy)_2_]^2+^ spectra is assigned to the phenanthroline ligand on the oligomer backbone. The 460 nm band in the A2[Ru(bpy)_2_]^2+^ spectrum corresponds to the MLCT transition (dπ(Ru) → π*), while in C989Ru, this transition is redshifted to 577 nm. As with A1, the presence of the 460 nm band in A2 (which lacks ruthenium) suggests that an undetermined component in the oligomer backbone also contributes to this absorption. In the mixture of A2 + C989Ru, both the 460 nm and 577 nm bands are observed, indicating that part of the C989Ru interacts with A2 to form the 460 nm transition, while some free C989Ru remains, contributing to the 577 nm absorption. The disappearance of the 577 nm band in the spectrum of A2[Ru(bpy)_2_]^2+^ suggests that all C989Ru in this complex is fully bound to A2.

### 2.3. Fluorescence Studies

**Figure 2:**
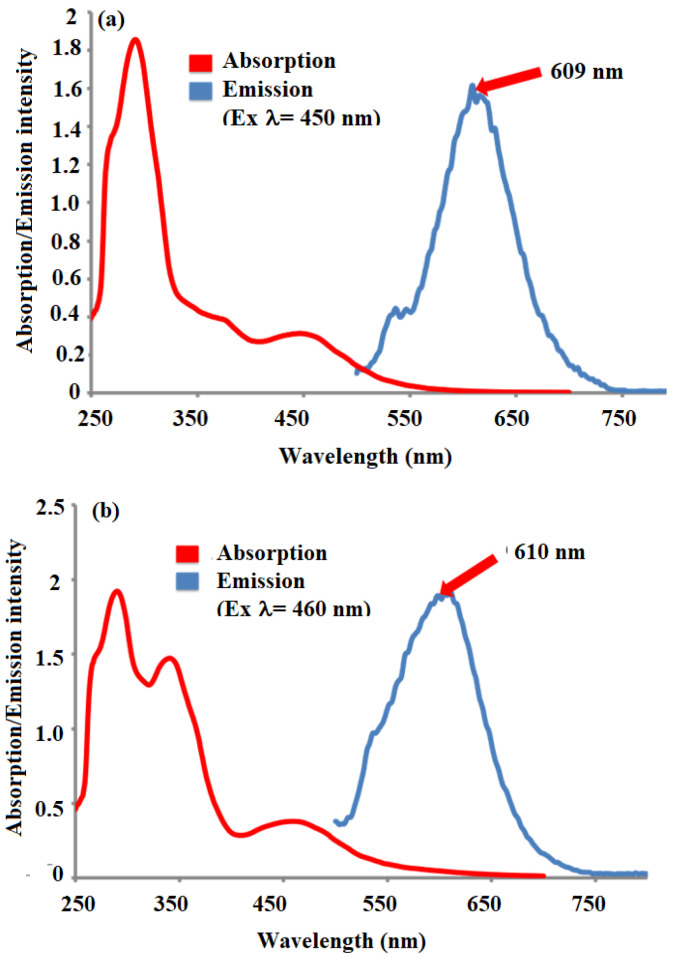
Absorbance and emission spectra of (**a**) A1[Ru(bpy)_2_]^2+^ and (**b**) A2[Ru(bpy)_2_]^2+^.

The emission spectrum was measured at room temperature in DMF using a 450 nm excitation light, with a quartz cell with a 1 cm path length, on an Agilent Cary Eclipse fluorescence spectrophotometer. An overlay of the absorbance and emission spectra is shown in Figure 2a. A distinct emission band was observed at 609 nm. The optical bandgap of A1[Ru(bpy)_2_]^2+^ was calculated to be 2.21 eV at 560 nm, where λ represents the band edge in the absorption spectrum. This value of 2.21 eV is consistent with a similar structure (8b) reported in the literature, which has an optical bandgap of 2.12 eV. A summary of the electro-optical data is provided in Table 1. Similarly, the emission spectrum for A2[Ru(bpy)_2_]^2+^ was collected at room temperature in DMF using the same experimental setup. An overlay of the absorbance and emission spectra is presented in Figure 2b, where an emission band is observed at 610 nm. The optical bandgap of A2[Ru(bpy)_2_]^2+^ was calculated to be 1.98 eV at 624 nm, with λ defined as the band edge in the absorption spectrum. This value of 1.98 eV aligns with the bandgap of 2.12 eV reported for a similar structure (8b) in the literature [19,20,21]. A summary of the electro-optical properties is shown in Table 2. One advantage of our structure compared to 8b is the functionalized aldehyde group, which provides a site for the attachment of additional π-acceptors (such as reduced graphene oxide, RGO) to further tune the bandgap. A comparison of the two structures is shown in Figure 2b. A1[Ru(bpy)_2_]^2+^ has a narrower HOMO–LUMO gap (1.9 eV) and a smaller optical bandgap (2.21 eV), which suggests that its electronic states are more closely spaced. This is likely due to the ligand effects and possibly the electronic structure of the ruthenium center itself. The smaller E_1/2_(ox) and E_1/2_(red) separation for A1 suggests that its electronic states are closer to each other, leading to a lower bandgap. A2[Ru(bpy)_2_]^2+^ has an optical bandgap of 1.98 eV. The literature complex (8b) has an optical bandgap of 2.12 eV. Generally, the optical bandgap is influenced by both the HOMO–LUMO gap and other factors, such as the degree of electronic coupling, the environment around the molecule (solvent, temperature), and the specific transitions that occur in the system. The variation in bandgap values between these complexes is primarily influenced by differences in their ligand environments, the electronic properties of the ruthenium center, and structural differences that affect the distribution of electron density, the redox potentials, and the nature of electronic transitions.

### 2.4. Cyclic Voltammetry

**Figure 3:**
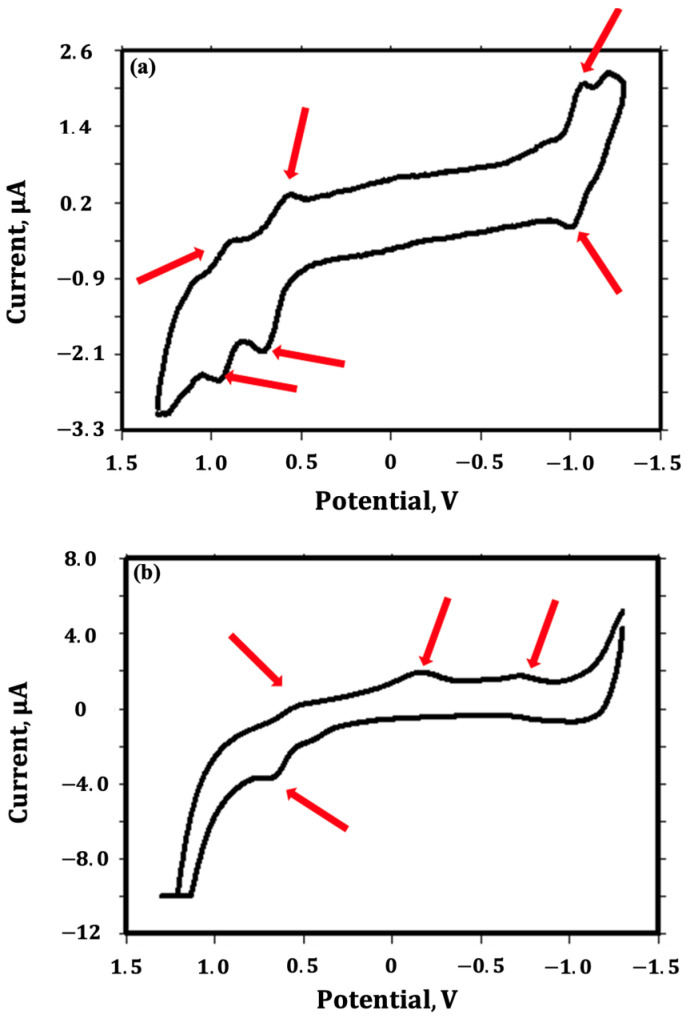
Cyclic voltammetry of (**a**) A1[Ru(bpy)_2_]^2+^ and (**b**) A2[Ru(bpy)_2_]^2+^ at a scan rate of 100 mV/s. The arrows displayed in the figure draw attention to the redox peaks discussed in the text.

The electrochemical redox potential of A1[Ru(bpy)_2_]^2+^ was determined by cyclic voltammetry (CV) using a three-electrode setup and a CV-50W electrochemical workstation. The working electrode was a glassy carbon electrode, the auxiliary electrode was a Pt wire, and the reference electrode was an Ag/Ag^+^ electrode. A 0.1 M solution of tetrabutylammonium hexafluorophosphate (TBAPF_6_) in acetonitrile served as the supporting electrolyte. The results are shown in Figure 3a,b. The first quasi-reversible oxidation wave, with an E_1/2_ of 0.6 V, corresponds to the Ru(II/III) couple. The second oxidation wave, with an E_1/2_ of 0.9 V, is attributed to the Ru(III/IV) couple. The reversible reduction wave at E_1/2_ = −1.0 V is assigned to a one-electron reduction process involving the bipyridine ligand [22]. To estimate the HOMO and LUMO energies, the oxidation and reduction potentials were used along with the known absolute HOMO energy of ferrocene (−4.8 eV). The HOMO energy of A1[Ru(bpy)_2_]^2+^ was calculated using the onset potential for the metal-based oxidation process at E_1/2_ = 0.9 V, while the LUMO energy was determined from the onset potential of the reduction process at E_1/2_ = −1.0 V. The calculated HOMO–LUMO gap is 1.9 eV, which is slightly smaller than the 2.34 eV gap reported for a similar structure in the literature [19,20,21]. This difference of 0.31 eV is likely due to the exciton binding energy of the oligomer. A summary of the electrochemical data is presented in Table 1. For A2[Ru(bpy)_2_]^2+^, Figure 3b, the electrochemical redox potential was measured under the same conditions using cyclic voltammetry. The first quasi-reversible oxidation wave at E_1/2_ = 0.6 V is assigned to the Ru(II/III) couple. The reduction waves observed at E = −0.2 V and E = −0.6 V are assigned to the reduction of the carbazole-based pendant and the bipyridine ligand, respectively. The HOMO energy was calculated from the oxidation potential, again referencing the HOMO level of ferrocene (−4.8 eV). A summary of the electrochemical data for A2[Ru(bpy)_2_]^2+^ is presented in Table 2. The calculated bandgap for A2[Ru(bpy)_2_]^2+^ is consistent with other reports, falling within the range of 0.9 eV to 2.4 eV, which is suitable for facilitating electron injection into the conduction band of reduced graphene oxide (RGO) [23]. The half-wave potential (E_1/2_) of 0.6 V determined is typical for ruthenium complexes with bipyridyl (bpy) ligands and indicates that the oxidation process is somewhat reversible but likely involves some degree of reorganization at the metal center during the redox transition. This redox couple is important because it reflects the stability of the Ru^2+^/Ru³^+^ couple, which governs the electron transfer characteristics of the complex. The calculated bandgap of A2[Ru(bpy)_2_]^2+^, which falls within the range of 0.9 eV to 2.4 eV, is ideal for facilitating electron injection into RGO because the HOMO and LUMO levels of A2[Ru(bpy)_2_]^2+^ align well with the conduction band of RGO. This makes the complex a good candidate for photoinduced charge transfer applications, where the excited-state ruthenium complex can transfer an electron to the reduced graphene oxide, enabling applications in solar cells, photocatalysis, and energy storage.

CV measures electrochemical transitions (oxidation and reduction of the material), which are related to charge transfer processes between the material and the electrode. These transitions typically reflect the energetic difference between the HOMO and LUMO levels and are often associated with electron transfer rather than photon absorption. UV–visible spectroscopy, on the other hand, measures optical transitions, where electrons in the valence band (or HOMO) are excited by photons to the conduction band (or LUMO). These transitions are influenced by the absorption of light, which can be affected by factors such as exciton binding energy (especially in organic materials) and optical selection rules. In molecular systems, excited-state dynamics can also influence the observed optical absorption. The electrochemical bandgap measured by CV, on the other hand, is more of a single-particle measure, reflecting the energy difference between individual molecular orbitals. In organic materials, CV is often used to measure charge injection and electronic transport, while UV–visible spectroscopy can measure optical excitations and is more sensitive to exciton binding. Organic semiconductors often show a larger difference between electrochemical and optical bandgaps due to the presence of excitons in the optical absorption process. In inorganic semiconductors (such as graphene oxide or metal oxides), the electrochemical and optical bandgaps may be closer but still show some differences due to correlation effects in the electronic structure. The differences in these bandgap values highlight the distinct ways these techniques probe the electronic properties of a material, with CV being more sensitive to charge transfer properties and UV–visible spectroscopy probing the material’s response to light and electron–hole pair formation.

### 2.5. Fluorescence Characteristics 

**Figure 4:**
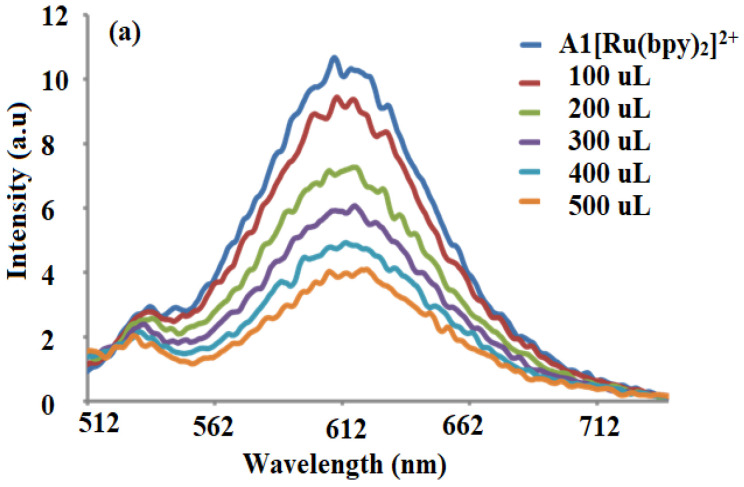

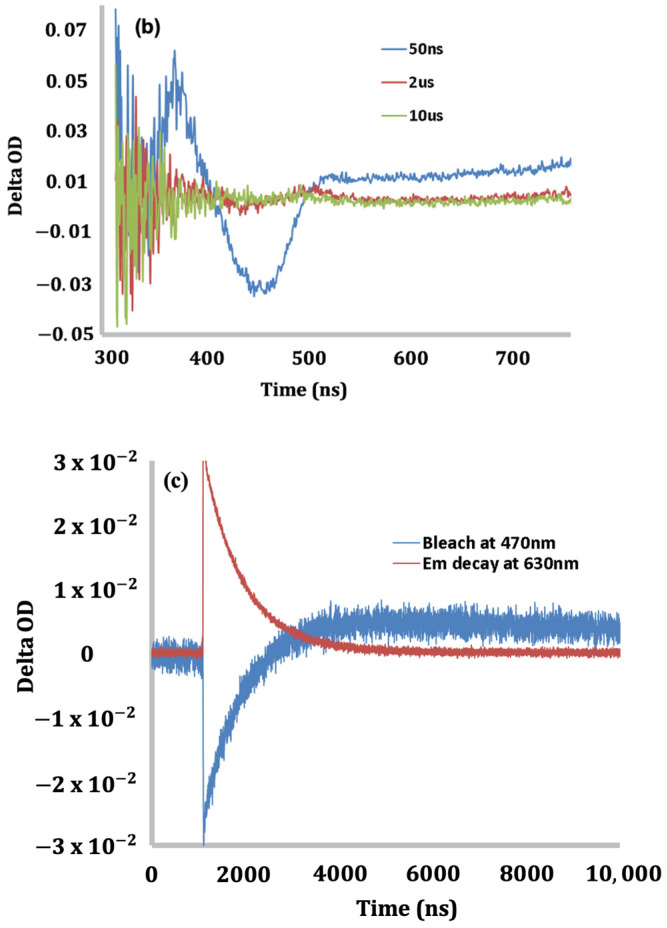
(**a**) Fluorescence emission quenching of A1[Ru(bpy)_2_]^2+^ by RGO. (**b**) Transient absorption spectrum of A2[Ru(bpy)_2_]^2+^ at 50 ns, 2 us, and 10 us. (**c**) Time-dependent emission decay for A2[Ru(bpy)_2_]^2+^ at 630 nm and bleaching at 470 nm.

To investigate the electrostatic interaction between A1[Ru(bpy)_2_]^2+^ and reduced graphene oxide (RGO), the two compounds were mixed together in DMF at varying concentrations of RGO from a stock solution (3 mg/10 mL). The resulting emission spectra were recorded, and the combined data are presented in Figure 4a. In control experiments, GO and rGO emission spectra displayed a broad peak at 577 nm, which is consistent with results reported in the literature [24]. Upon the addition of 500 µL of RGO, a significant quenching of the fluorescence emission was observed compared to the initial A1[Ru(bpy)_2_]^2+^ spectrum. This quenching suggests an interaction between the electron density of A1[Ru(bpy)_2_]^2+^ and RGO, supporting the presence of a π–π interaction between the two. The fluorescence quenching observed aligns with similar results reported for electron donors interacting with reduced graphene oxide [24,25]. The fluorescence quenching can occur through a π–π stacking interaction between the aromatic rings of the bpy ligands in A1[Ru(bpy)_2_]^2+^ and the conjugated π-system in RGO. These interactions can facilitate non-radiative energy transfer from the excited state of the ruthenium complex to the RGO. When the excited A1[Ru(bpy)_2_]^2+^ complex interacts with the RGO, the energy of the excited state may be transferred to the RGO via these π–π interactions, thus deactivating the excited state without the emission of light, resulting in fluorescence quenching. The fluorescence quenching can also occur due to electron transfer between the HOMO of A1[Ru(bpy)_2_]^2+^ and the π-system of RGO. In the presence of RGO, the excited-state electron in A1[Ru(bpy)_2_]^2+^ (which has a relatively high-lying HOMO, around −5.7 eV) may be transferred to the conducting π-band of RGO (which has a relatively low-lying conduction band). This photoinduced electron transfer process competes with the radiative recombination (fluorescence), leading to a reduction in fluorescence intensity. The RGO serves as an electron acceptor or energy sink, facilitating non-radiative decay of the excited state of the ruthenium complex, thereby quenching the fluorescence. The concentration dependence of the quenching suggests that the interaction between A1[Ru(bpy)_2_]^2+^ and RGO is intensity-dependent, further supporting the idea of an electrostatic or charge transfer interaction between the two species.

RGO acts as an electron acceptor, accepting electrons from the excited complex, which results in the decay of the excited state without the emission of light. This quenching is often attributed to charge transfer interactions, where the excited state of the complex is quenched due to the movement of an electron from the A1[Ru(bpy)_2_]^2+^ to the RGO surface. In summary, we have successfully prepared a nanohybrid material consisting of A1[Ru(bpy)_2_]^2+^ and RGO through a non-covalent electrostatic method. Fluorescence quenching studies, UV-Vis spectroscopy, and cyclic voltammetry confirm the existence of a π–π interaction between the conjugated ruthenium(II) polypyridyl-based oligomer (A1[Ru(bpy)_2_]^2+^) and RGO. The optical bandgap of A1[Ru(bpy)_2_]^2+^ was determined to be 2.21 eV, while the bandgap obtained from cyclic voltammetry was 1.9 eV. These findings suggest that A1[Ru(bpy)_2_]^2+^ can facilitate the injection of electrons into the conduction band of RGO.

The transient absorption measurements shown in Figure 4b were performed using nanosecond laser pulses from a Spectra-Physics Quanta-Ray Lab-170 Nd laser, coupled with a Versa Scan OPO (tuned to 460 nm, full width at half maximum ~5−7 ns, repetition rate = 1 Hz). These were integrated into an Edinburgh Instruments LP920 system, which was equipped with a xenon light source. Detection was achieved either by a gated CCD (Princeton Instruments PI-MAX3) or a Hamamatsu R928P PMT, with signal acquisition handled by a Tektronix TDS 3032C digital phosphor oscilloscope. The system was electronically synchronized using the Edinburgh Instruments F900 software. A total of 180 laser shots were averaged for the transient absorption spectra shown in Figure 4b. The transient absorption spectra obtained were consistent with previously published data for similar [Ru(bpy)_3_]^2+^ derivatives [12,26,27]. The dominant excited-state transitions observed around 360 nm correspond to the ground-state absorption of reduced bipyridine [12]. A ground-state bleach is seen at 450 nm, followed by a positive transient absorption. No significant ground-state absorption is detected above 520 nm, indicating the absence of long-lived states in that region. For the emission decay studies on A2[Ru(bpy)_2_]^2+^, time-correlated single-photon counting (TCSPC) was employed, as shown in Figure 4c. These measurements were performed using a high-power laser pulse, which likely led to a decay process involving annihilation. The emission decay at 630 nm and bleaching at 470 nm for A2[Ru(bpy)_2_]^2+^ are shown in Figure 4c. In comparison to the kinetic trace of [Ru(bpy)_3_]^2+^ reported by [28], the bleaching of A2[Ru(bpy)_2_]^2+^ exhibited a higher baseline compared to the decay. This suggests the involvement of an additional energy process, possibly electron transfer, which could be influencing the observed dynamics.

### 2.6. Raman, SEM, and XPS Studies 

**Figure 5:**
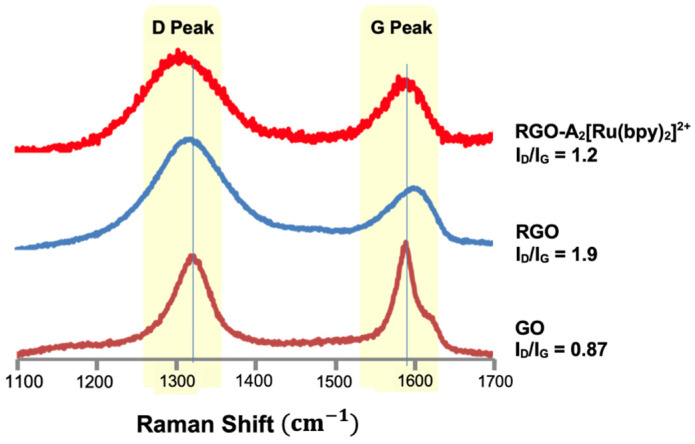
Raman spectra of GO, RGO, and RGO-A2[Ru(bpy)_2_]^2+^.

The Raman spectra of GO, RGO, and RGO-A2[Ru(bpy)_2_]^2+^ are shown in Figure 5. The samples were prepared by drop casting a stock solution onto a silicon wafer, which was allowed to dry for approximately 30 min. Spectra were then obtained using a 785 nm excitation laser attached to a Renishaw Ramascope instrument, coupled with an Olympus BH2-UMA microscope. The D and G peaks were analyzed, and the ratio of the intensities (I_D_/I_G_) was used to evaluate the degree of functionalization on the surface. GO displayed the characteristic D and G bands, centered at 1342 and 1581 cm^−1^, respectively. GO-to-RGO transformation was accompanied by surface defects and the appearance of an increasing number of sp2 hybridized C=C carbons. This correlated with an increasing I_D_/I_G_ ratio of 1.9 as the intensity of the D band increased, while the G band decreased and blueshifted in comparison to GO (I_D_/I_G_ = 0.87). In RGO-A2[Ru(bpy)_2_]^2+^, the G band redshifted to lower wavenumber regions similar to those observed in GO. This indicated chemical bond formation between RGO and A2[Ru(bpy)_2_]^2+^. In our study, the lower I_D_/I_G_ ratio of 1.2 for the polymer-grafted RGO compared to RGO indicates a self-healing effect that has been observed in the literature when the defect sites of RGO are functionalized with polymer [9]. This result provides indirect evidence that covalent attachment between A2[Ru(bpy)_2_]^2+^ and RGO exists. 

**Figure 6:**
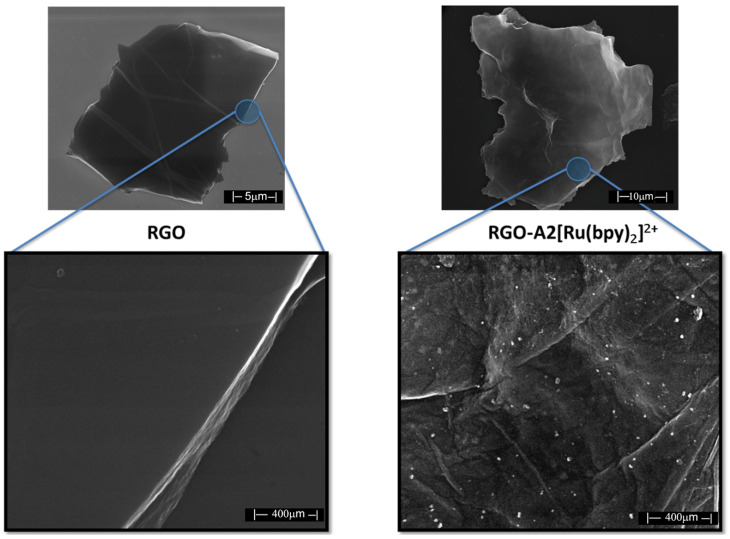
Scanning electron microscope (SEM) images of RGO and RGO-A2[Ru(bpy)_2_]^2+^. Note the increased surface roughness observed in RGO-A2[Ru(bpy)_2_]^2+^ due to the formation of a thin film of the oligomer material that is covalently attached to the RGO surface.

The surface of reduced graphene oxide (RGO) was further examined using scanning electron microscopy (SEM) to obtain a visual image of its morphology, as shown in Figure 6. A dilute solution of the sample was deposited onto a silicon wafer, which was then analyzed using a Nova Nano SEM 600 instrument. The SEM images of the initial RGO surface and the RGO-A2[Ru(bpy)_2_]^2+^ composite are shown in Figure 6. The image of the initial RGO surface reveals a relatively smooth texture with a lateral length of approximately 12 μm. In contrast, the surface of RGO-A2[Ru(bpy)_2_]^2+^ appears rougher, while maintaining a similar lateral length. The increased surface roughness observed in RGO-A2[Ru(bpy)_2_]^2+^ is consistent with other reports of polymer/graphene composites in the literature [11,12], suggesting the formation of a thin film of the oligomer material that is covalently attached to the RGO surface.

### 2.7. X-Ray Photoelectron Spectroscopy

**Figure 7:**
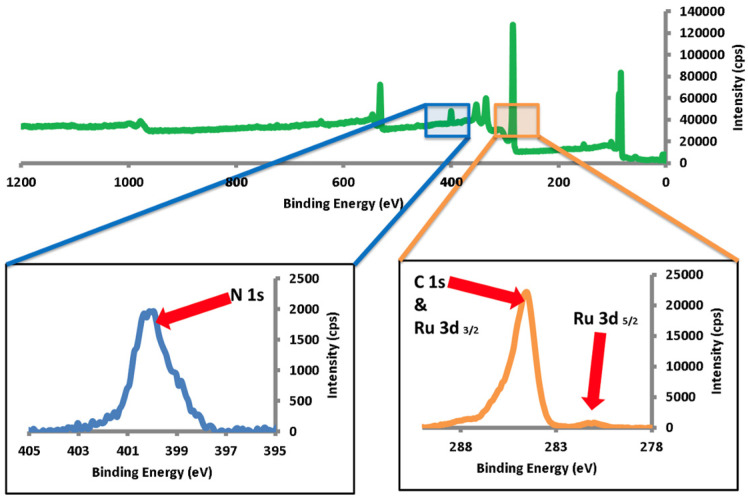
X-ray photoelectron spectroscopy (XPS) spectrum of RGO-A2[Ru(bpy)_2_]^2+^.

RGO-A2[Ru(bpy)_2_]^2+^ was further characterized using X-ray photoelectron spectroscopy (XPS) to quantitatively assess the elemental composition of its surface. The XPS results are shown in Figure 7. The high-resolution spectra confirm the presence of elemental ruthenium and nitrogen. Specifically, the Ru 3d_5_/_2_ peak is observed at 281 eV, and the N 1s peak appears at 400 eV. The Ru 3d_3_/_2_ peak overlaps with the larger C 1s peak, which is typical in such analyses. These values are consistent with those reported in the literature for elemental ruthenium and nitrogen [29,30]. The calculated N ratio of 16:1 suggests that the ruthenium complex is loaded at a 1:2 ratio, indicating a partial loading of ruthenium on the surface.

### 2.8. Computational Studies 

**Figure 8:**
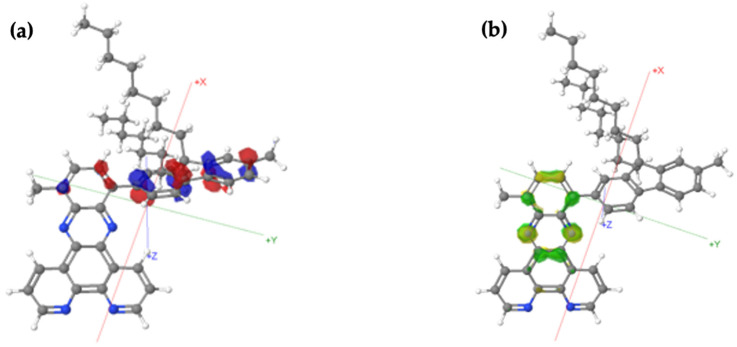

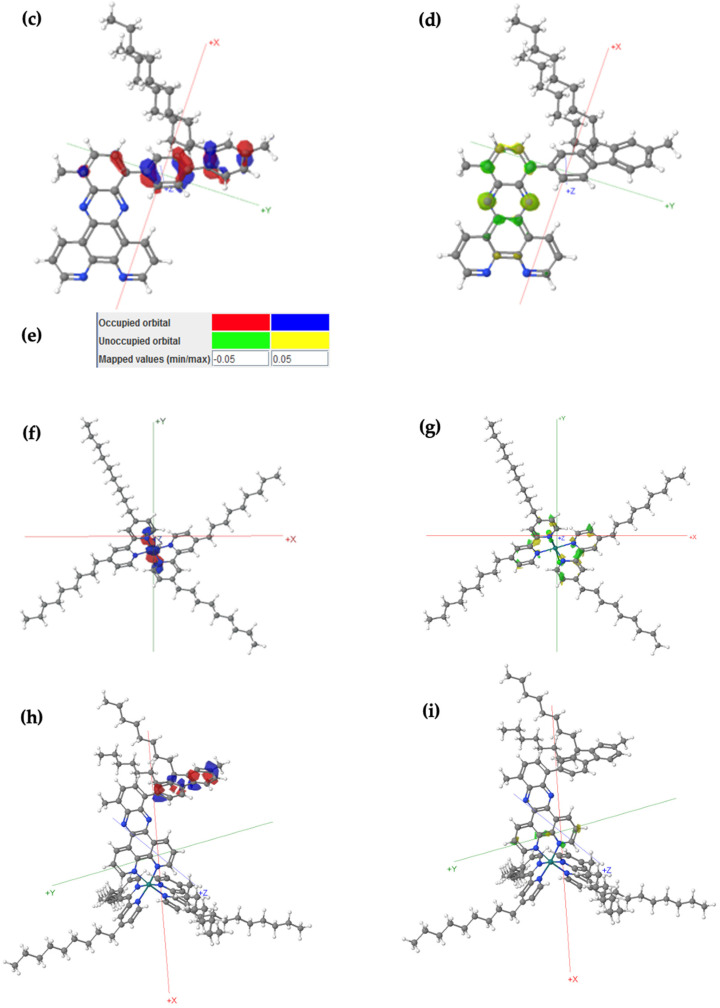

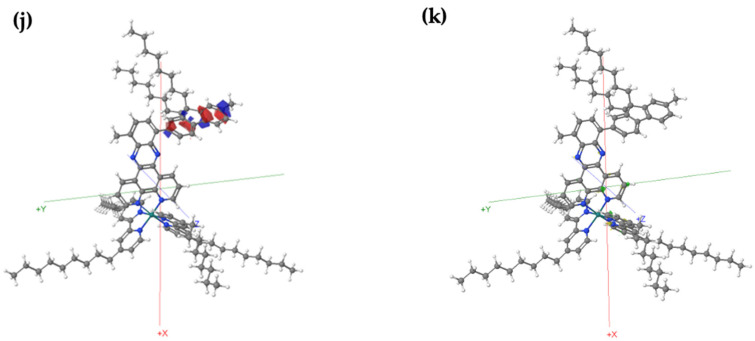
Relaxed geometries of A1 monomer backbone with HOMO orbitals followed by the LUMO orbitals for each: (**a**,**b**) FHI-aims HF; (**c**,**d**) FHI-aims PBE/PBE0. (**e**) Shows the color scale and values for the orbitals in eVs. Relaxed geometries of C989Ru chromophore with HOMO orbitals followed by the LUMO orbitals for each: (**f**,**g**) Gaussian via HF. Relaxed geometries of A1[Ru]bpy monomer with HOMO orbitals followed by the LUMO orbitals for each: (**h**,**i**) FHI-aims via PBE/PBE0 (**j**,**k**); FHI-aims (PBE/PBE0) with van der Waals force considerations.

**Figure 9:**
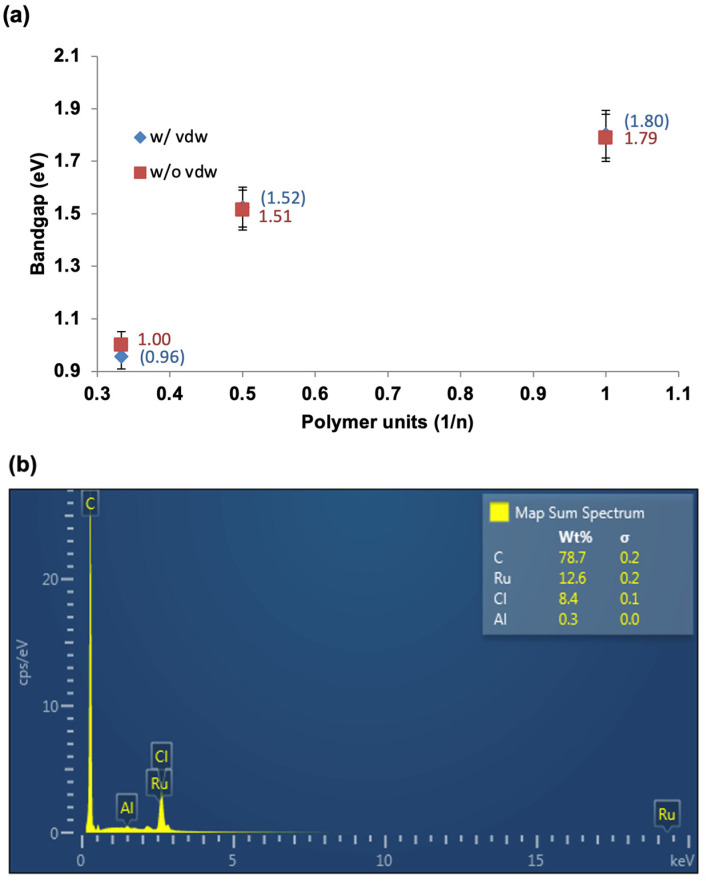
(**a**) Extrapolated data of A1[Ru]bpy^2+^ per unit length. Blue graph includes van der Waals considerations, while the red graph does not. (**b**) EDX data reveal the element concentration of A1[Ru] powder.

The first step in calculating the relaxed geometry of the A1[Ru] complex monomer involved relaxing the backbone, denoted as A1. FHI-aims simulations were carried out using light settings in all cases, and the results are shown in Figure 8a–e. The resulting bandgap and energies are summarized in Table S1 of the Supporting Information, which lists the values obtained using the PBE functional. These results are consistent with similar structures reported in the literature [1,2], where the bandgap typically ranges from 2.5 eV to 3.5 eV, supporting the soundness of our calculations. Next, the relaxation of the C989Ru complex, depicted in Figure 8f-g, was performed to understand the ground-state conformation of the hybrid complex. The bond lengths are presented in Table S2 of the Supporting Information, along with the corresponding bandgap and total energies. The Hartree–Fock (HF) method failed to converge, suggesting that HF is not suitable for geometric optimization of this chromophore. Therefore, the PBE method was employed, as shown in Figure 8f–g. The resulting bond lengths are in good agreement with published values [3]. After optimizing the geometries of both the backbone and the C989Ru cap, the next step involved attaching the C989Ru cap to the backbone matrix and performing a relaxation simulation of the resultant geometry. The achieved geometric conformation for the monomer units is shown in Figure 8h–k. While the bandgap characteristics of the monomer are not yet fully known, data previously obtained in our lab indicate that the bandgap for the polymer is 2.21 eV, while the HOMO–LUMO gap for A1[Ru] is 1.9 eV, as shown in Table S3 of the Supporting Information. Using this data, we hypothesized that the monomer should exhibit a bandgap larger than 1.9 eV, ideally close to or greater than this value, while minimizing the total energy. The results from these calculations are summarized in Table S4 of the Supporting Information. Based on the geometry, we observed that the chains are linear in structure, forming an octahedral configuration. PBE/PBE0 calculations provided more-accurate results, leading to the decision to continue calculations using PBE/PBE0 theory exclusively. To further improve accuracy, van der Waals forces were incorporated into the simulations, as shown in Table S5. Comparing the FHI-aims results with and without these corrections (see Table S6) suggests that including van der Waals interactions can enhance the accuracy of the calculations. Therefore, we decided to continue the calculations with these settings. The extrapolated bandgap values are displayed in Figure 9a. The dimer yielded a bandgap of 1.525 eV, while the trimer had a bandgap of 0.957 eV. Based on previous data from Table S3, we expect the bandgap of the polymer to be around 1.9 eV, which aligns with our findings.

The confirmed bandgaps from our simulations still deviate significantly from the measured values for the polymer, which may be attributed to the positive charge of the polymer. In real-world conditions, the polymer is likely to contain one or more counterions to balance the positive charge of the molecule. Energy-dispersive X-ray spectroscopy (EDX) data, shown in Figure 9b, reveal a notable presence of chlorine in the A1[Ru] powder. This suggests that A1[Ru] utilizes chloride ions to fulfill its counter-ionic requirements. Therefore, it is reasonable to conclude that once the chromophore is incorporated into the polymer, the chlorine ions interact with the resulting molecule, potentially influencing its electronic properties and affecting the bandgap.

**Figure 10:**
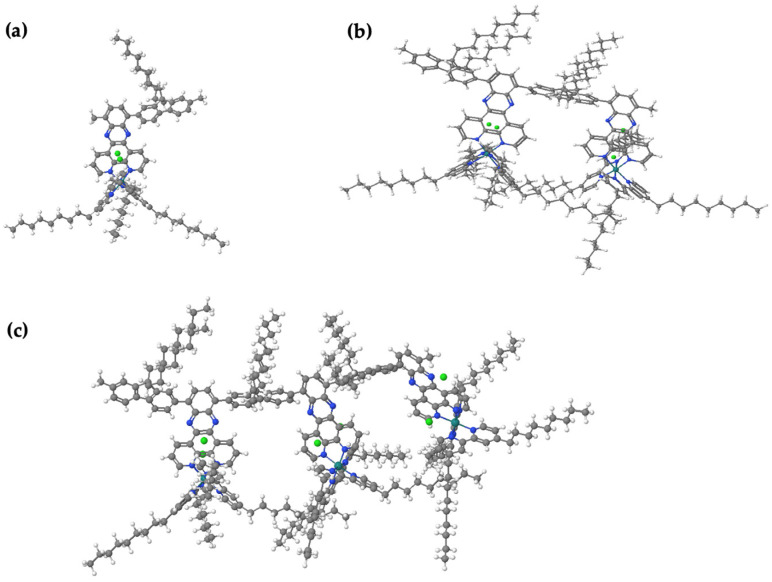
Geometric conformation of [A1[Ru]bpy^2+^]2Cl^−^ for the (**a**) monomer, (**b**) dimer, and (**c**) trimer.

**Figure 11:**
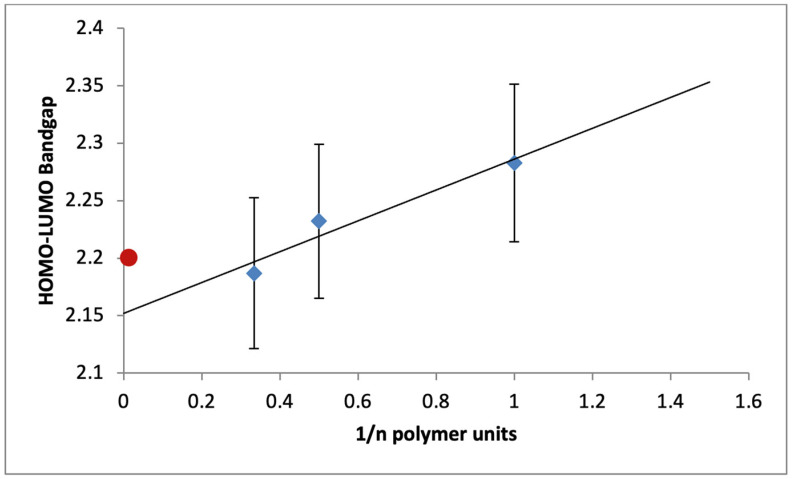
Extrapolated bandgaps of [A1[Ru]bpy^2+^]_2_Cl^−^ where the diamonds represent the three data points obtained from theoretical calculations. The circle signifies the bandgap of P3HT (2.2 eV) for comparison purposes.

Calculations with chlorines electrostatically interacting with the polymer units were then run, as shown in Figure 10a–c. The results yield bandgaps far closer to the experimental data than previous calculations, as seen in Figure 11 and Table 2. Thus, with the inclusion of chlorines in the matrix, we were able to achieve similar results, even though the values were a bit higher. This trend has been reported previously for other ruthenium polypyridyl complexes [31].

## 3. Materials and Methods

All reagents were purchased from Fisher Scientific (Raleigh, NC, USA) and used as received unless otherwise stated. Proton nuclear magnetic resonance spectra (^1^H NMR) were recorded on a Varian (500 MHz) spectrophotometer. Chemical shifts δ were reported in parts per million (ppm) units, with tetramethyl silane (TMS at 0.0 ppm) as an internal reference. All reagents were purchased from Fisher Scientific and used as received unless otherwise stated. C642 is commercially available and was purchased from Fisher Scientific. Proton nuclear magnetic resonance spectra (^1^H NMR) were recorded on a Varian (500 MHz) spectrophotometer. Chemical shifts δ were reported in parts per million (ppm) units with tetramethyl silane (TMS at 0.0 ppm) as an internal reference. All reagents were purchased from Fisher Scientific and used as received unless otherwise stated.

### 3.1. Synthesis of Graphene Oxide (GO)

A mixture of graphite (5 g), NaNO_3_ (5 g), and H_2_SO_4_ (230 mL) was stirred in an ice bath. KMnO4 (15 g) was slowly added while stirring. The rate of addition was controlled to prevent the reaction temperature from exceeding 20 °C. The mixture was then transferred to a 35 °C oil bath and stirred for about 30 min, forming a thick paste. Subsequently, deionized water (130 mL) was added gradually, causing an increase in temperature. After 15 min, the mixture was further treated with de-ionized water (700 mL) and 30% H_2_O_2_ solution (50 mL). The warm solution was then filtered and washed with deionized water until neutralized (pH 7) and dried under vacuum.

### 3.2. Synthesis of Reduced Graphene Oxide (RGO) by Sodium Borohydride

GO (1 g) was placed in a 500 mL round-bottom flask, which was followed by the addition of deionized water (250 mL). After stirring and ultra-sonication for 30 min, sodium borohydride (5 g) was gradually added to the solution. Sodium borohydride was chosen for its selective, efficient, and safe reduction of GO to rGO, which leads to significant improvements in electrical conductivity, while still allowing for tunable surface chemistry. Sodium borohydride reduction reduces many oxygen-containing groups and causes partial reduction where the surface of rGO can still undergo chemical functionalization, making rGO suitable for applications like sensors, batteries, and supercapacitors, where specific surface interactions are needed.

The mixture was stirred for 4 h at room temperature and then filtered. The filter cake was washed with deionized water until the filtrate was clear. The remaining black solid was dried under vacuum.

### 3.3. Synthesis of 4,7-Dibromobenzo[1,2,5]Thiadiazole (1)

A mixture of 1,2,5-benzothiadiazole (20.0 g) in aq. HBr (48%, 60 mL) was heated to reflux with stirring, while Br_2_ (22.6 mL) was added slowly over 1 h. Towards the end of the addition, the mixture became a suspension. To facilitate stirring, aq. HBr (48%, 40 mL) was added. The mixture was heated to reflux for an additional 2 h then filtered while hot, cooled, filtered again, and washed well with deionized water. The compound was dried over Na_2_SO_4_ and recrystallized with MeOH to give a white needle product (38.0 g, 88%). ^1^H NMR (500 MHz, chloroform-d) δ 7.75–7.73 (s, 3H).

### 3.4. Synthesis of 3,6-Dibromobenzene-1,2-Diamine (2)

To a suspension of 1 (5.0 g) in EtOH (170 mL), NaBH4 (11.4 g) was added portionwise at 0 °C. The mixture was then stirred for 20 h at room temperature. Afterwards, the solvent was removed under vacuum and deionized water (100 mL) was added. The mixture was extracted with Et_2_O. The organic phase was washed with saturated aq. NaCl solution and dried over Na_2_SO_4_. The compound was then dried under vacuum to give a white solid product (3.9 g, 87%). ^1^H NMR (500 MHz, chloroform-d) δ 6.97–6.76 (s, 2H), 3.94–3.80 (s, 4H).

### 3.5. Synthesis of 1,10-Phenanthroline-5,6-Dione (3)

Concentrated H_2_SO_4_ (20 mL) and nitric acid (10 mL) were added dropwise to a mixture of 1,10-phenanthroline (1.00 g) and KBr (5.95 g) at 0 °C. The mixture was refluxed at 80 °C for 2 h and then cooled to room temperature. The contents of the reaction flask were diluted with deionized water (400 mL) and neutralized with sodium bicarbonate (NaHCO_3_). The product was extracted with methylene chloride and dried over anhydrous MgSO_4_. After all solvents were removed under vacuum, the compound was recrystallized with MeOH to give a yellow solid (1.1 g, 95%). 1H NMR (500 MHz, chloroform-d) δ 9.25–9.08 (s, 2H), 8.61–8.33 (m, 2H), 7.62–7.58 (s, 2H).

### 3.6. Synthesis of 10,13-Dibromodipyrido[3,2-a:2,3-c]Phenazine (C440)

A mixture of 2 (0.53 g) and 3 (0.42 g) in EtOH (25 mL) was refluxed and stirred for 3 h. The reaction mixture was allowed to cool to room temperature, vacuum filtered, and then washed with (CH_3_)_2_CO (20 mL) and Et2O (40 mL) to give a yellow solid (0.364 g, 91%). ^1^H NMR (500 MHz, chloroform-d) δ 9.79–9.67 (s, 2H), 9.37–9.27 (s, 2H), 8.16–8.05 (s, 2H), 7.89–7.79 (s, 2H).

### 3.7. Synthesis of A1

A mixture of C440 (100 mg), C642 (100 mg), Pd(PPh_3_)_4_ (10 mg), and Cs_2_CO_3_ (276 mg) in DMF (4 mL) was stirred at 120 °C for 36 h under dry nitrogen. The reaction mixture was allowed to cool to room temperature then precipitated in methanol (MeOH). The collected solid was washed with MeOH and deionized water, then with MeOH again. The crude product was extracted with acetone (200 mL) for 3 days in a Soxhlet apparatus to remove impurities. The residual solid in the thimble was dried under vacuum to give an orange solid (132 mg). ^1^H NMR (500 MHz, Chloroform-d) δ 9.03–8.94 (ddd, 3H), 8.88–8.82 (dd, 1H), 8.30–8.24 (dd, 1H), 8.09–8.02 (m, 2H), 8.01–7.82 (m, 4H), 7.74–7.68 (t, 2H), 7.66–7.60 (dd, 1H), 7.58–7.50 (td, 1H), 7.50–7.43 (td, 1H), 2.19–2.12 (m, 2H), 1.98–1.91 (m, 2H), 1.53–1.44 (m, 4H), 1.38–1.21 (m, 21H), 0.90–0.82 (m, 6H).

### 3.8. Synthesis of C989Ru

A mixture of RuCl_3_·3H_2_O (84 mg), LiCl (92 mg), and 4,4′-Dinonyl-2,2′-dipyridyl (262 mg) in DMF (5 mL) was heated to 120 °C for 8 h under dry nitrogen. After the removal of the solvent in vacuum, the resulting purple solid was dissolved in DCM and washed with deionized water (2 × 50 mL). After the evaporation of the DCM, the resulting solid was washed with hexane (4 × 40 mL) upon sonication. Subsequent removal of the hexane resulted in a purple solid (200 mg). ^1^H NMR (500 MHz, Chloroform-d) δ 8.67–8.63 (d, 1H), 8.63–8.57 (d, 1H), 7.22–7.16 (dd, 1H), 3.43–3.37 (t, 2H), 1.65–1.57 (q, 2H), 1.30–1.22 (m, 12H), 0.90–0.82 (m, 3H).

### 3.9. Synthesis of A1[Ru(bpy)_2_]^2+^

A mixture of A1 and C989Ru in EtOH (5 mL) and deionized water (5 mL) was refluxed overnight under dry nitrogen. The reaction mixture was cooled to room temperature and the solvent was evaporated. The residue was then suspended in EtOH and filtered. The filtrate was precipitated in MeOH, and the solid was collected and dried under vacuum (384 mg). ^1^H NMR (500 MHz, chloroform-d) δ 8.67–8.63 (d, 1H), 8.63–8.57 (d, 1H), 7.22–7.16 (dd, 1H), 3.43–3.37 (t, 2H), 1.86–1.80 (t, 1H), 1.65–1.57 (q, 2H), 1.35–1.20 (m, 17H), 0.90–0.82 (m, 4H).

### 3.10. Synthesis of 3,6-Dibromocarbazole (4)

To a solution of carbazole (10 g) in acetic acid and sodium acetate buffer solution, bromine (7.5 mL) was added dropwise at 0 °C and stirred for 4 h. Afterward, a 2M NaOH aqueous solution was poured into the reaction mixture. The crude product was filtered and washed with deionized water and then recrystallized from ethanol, resulting in a white solid (15.4 g, 79%). ^1^H NMR (500 MHz, Chloroform-d) δ 8.12–8.08 (d, 2H), 7.39–7.29 (m, 3H), 7.28–7.24 (s, 1H).

### 3.11. Synthesis of C429

A mixture of compound 4 (8.4 g) and potassium tert-butoxide (5.5 g) in anhydrous DMF (200 mL) was heated at 110 °C for 30 min, then 4-fluorobenzaldehyde (7 mL) was added dropwise to the solution, followed by heating and stirring for an additional 36 h. The mixture was then cooled to room temperature and poured into ice water, then filtered and dried. The purified yellow product was obtained after recrystallization from acetone/water. ^1^H NMR (500 MHz, Chloroform-d) δ 10.20–10.09 (m, 1H), 8.26–8.00 (m, 4H), 7.80–7.67 (dd, 2H), 7.59–7.50 (dq, 2H), 7.39–7.29 (m, 2H).

### 3.12. Synthesis of A2

A mixture of C440 (100 mg), C642 (100 mg), C429 (97 mg), Pd(PPh_3_)_4_ (10 mg), and Cs_2_CO_3_ (276 mg) in 4 mL DMF was stirred at 120 °C for 36 h under dry nitrogen. The reaction mixture was allowed to cool to room temperature and then precipitated in MeOH. The collected solid was washed with MeOH and deionized water, then with MeOH again. The collected crude product was extracted with acetone (200 mL) for 3 days in a Soxhlet apparatus to remove impurities. The residual was dried under vacuum to give an orange solid (143 mg). ^1^H NMR (500 MHz, Chloroform-d) δ 9.00–8.94 (dd, 1H), 8.26–8.17 (m, 2H), 8.15–8.07 (m, 2H), 8.05–7.95 (m, 2H), 7.94–7.83 (m, 2H), 7.80–7.68 (m, 2H), 3.82–3.75 (t, 1H), 3.26–3.16 (p, 2H), 2.95–2.88 (t, 1H), 2.27–2.20 (m, 1H), 2.11–2.04 (m, 1H), 1.65–1.53 (m, 3H), 1.44–1.34 (m, 2H), 1.31–1.21 (m, 17H), 0.90–0.82 (m, 6H).

### 3.13. Synthesis of A2[Ru(bpy)_2_]^2+^

A mixture of A2 and C989Ru in EtOH (5 mL) and H_2_O (5 mL) was refluxed overnight under dry nitrogen. The reaction mixture was cooled to room temperature and the solvent was evaporated. The residue was then suspended in EtOH and filtered. The filtrate was then precipitated in MeOH, and the solid was collected and dried under vacuum (150 mg). 1H NMR (500 MHz, chloroform-d) δ 8.67–8.63 (d, 1H), 8.63–8.57 (d, 1H), 8.10–8.04 (d, 1H), 7.91–7.81 (m, 2H), 7.80–7.73 (ddd, 1H), 7.22–7.16 (dd, 1H), 3.43–3.37 (t, 2H), 1.86–1.80 (t, 2H), 1.65–1.57 (q, 2H), 1.36–1.20 (m, 24H), 0.90–0.82 (m, 6H).

### 3.14. Synthesis of RGO-A2[Ru(bpy)_2_]^2+^

A mixture of A2[Ru(bpy)_2_]^2+^ (100 mg), RGO (5 mg), and sarcosine (5 mg) in anhydrous DMF (50 mL) was refluxed for 72 h. Afterward, the mixture was cooled to room temperature and 100 mL of methanol was added. The crude product was collected by filtration using a polycarbonate film (0.45 μm), washed with ethanol and dichloromethane, and then dissolved in a small amount of DMF, before undergoing precipitation from methanol. The procedure was repeated at least three times. The gray-black solid obtained was dried under vacuum overnight.

### 3.15. Computational Methods

The calculations were performed using FHI-aims via Duke University’s computer cluster. FHI-aims uses a numeric atomic orbital (NAO) basis set, which is efficient and well-suited for large systems and periodic calculations. The NAO basis sets in FHI-aims are highly efficient because they are optimized for plane-wave expansion and do not require the explicit definition of Gaussian functions. They allow for a more systematic improvement in accuracy, with fewer computational resources for large systems. These studies were usually calculated under light relaxation settings with bfg of E-2, using either PBE or HF functions. PBE is a generalized gradient approximation (GGA) functional, widely used in DFT calculations for many materials and molecular systems. It is relatively efficient and provides a good balance between accuracy and computational cost. HF was chosen for its ability to provide a more rigorous treatment of the exchange interaction and is useful for validating the results obtained using PBE and PBE0. HF allowed the researchers to cross-check the results and ensure that the functional dependence did not significantly alter the outcome of the calculations. It is particularly suitable for systems with weak intermolecular interactions, such as van der Waals interactions or when researchers want to perform large-scale computations without sacrificing too much accuracy. PBE tends to underestimate the bandgap of semiconductors and systems with significant electron correlation, but it is still a widely used functional due to its efficiency. Geometric optimization was run first, followed by molecular orbital calculations on the relaxed molecule for both Gaussian and FHI-aims calculations. PBE functions were followed by single-point conformation conducted via PBE0. PBE0 was chosen for single-point energy calculations because of its improved accuracy for electronic structure properties (such as HOMO–LUMO gaps, ionization potentials, or electron affinities). In particular, PBE0 is known to give more-accurate electronic band gaps and is often used for systems where electronic properties are crucial. The HF functions were followed by single-point conformation conducted via HF again. Gaussian 09 is a more widely used software compared to the relatively new FHI-aims software (https://fhi-aims.org accessed on 12 May 2016), so HF and PBE calculations are run on both packages for comparison purposes. Gaussian typically uses Gaussian-type orbitals (GTOs), which are popular and well-established. GTOs are more compact in real space, making them more efficient for localized systems (especially small-to-medium-sized molecules) when compared to plane-wave-based approaches. The polymer properties for bandgaps were calculated by plotting results for oligomers with increasing chain length against 1/n, where n represents the number of monomer repeat units. Note that the minimum number of repeat units is three for a molecule to be considered a polymer. The data were extrapolated using second-order polynomial fits, and the extrapolating energy levels were calculated for monomers through trimers for A1[Ru].

### 3.16. Raman Analysis

A sample was prepared from a stock solution of GO or RGO in ethanol (EtOH) (3 mg/10 mL) and was drop cast onto a silicon wafer. The wafer was allowed to dry for approximately 30 min. Afterwards, a 785 nm excitation laser attached to a Renishaw Ramascope instrument with an Olympus BH2-UMA microscope was used to obtain the spectra. The typical Raman spectrum of graphene commonly exhibits two major bands defined as the D and G bands. The D band contributes to transformation of sp2 carbons to sp3 carbons due to covalent functionalization [32,33,34,35]. The average ratio of the D-to-G peak (ID/IG) was compared to determine the degree of functionalization on the surface.

### 3.17. Atomic Force Microscopy Studies

A topographic image of RGO was captured using tapping mode atomic force microscopy (AFM) on the model Nanoscope III, from Digital Instruments, Inc., Santa Barbara, CA, USA. The sample was prepared using a diluted stock solution of RGO in EtOH (1 mg/10 mL) and was drop cast onto a mica wafer. The wafer was allowed to dry for approximately 30 min.

### 3.18. XPS Studies

XPS analysis was carried out using a monochromatic microspot X-ray beam originating from the A1 Kα source with a spot diameter of ~600 μm from a Surface Science Instrument 100-06 spectrometer (Kratos Analytical Inc., Nanuet, NY, USA). The XPS data were analyzed with a least-square fitting routine and into individual Gaussian peaks, assuming a linear background over the energy range of the fit. Deconvolution was performed with the constraint of equal full width at half maximum (FWHM) value for the prominent lines of different spectra.

### 3.19. Scanning Electron Microscopy Studies

SEM was performed on a Nova NanoSEM 600 instrument, (Schaumburg, IL, USA) and the sample was prepared from a dilute solution of RGO (1 mg/10 mL) deposited onto a silicon wafer and examined.

## 4. Conclusions

We have successfully developed and outlined the synthetic strategies employed to obtain the novel conjugated oligomer A2[Ru(bpy)_2_]^2+^, a complex incorporating a Ru(II) polypyridyl coordination with 2,2′-bipyridine (bpy) ligands. The synthesis of this oligomer involved a series of steps, culminating in a carefully controlled reaction to ensure the desired structural integrity and electronic properties of the final product. A2[[Ru(bpy)_2_]^2+^ was systematically studied using a variety of spectroscopic and electrochemical techniques to evaluate its physical, electronic, and optoelectronic properties. The fluorescence quenching studies revealed strong evidence of electron transfer through the conjugated oligomer backbone. These findings suggest that the oligomer possesses good charge transport characteristics, which are critical for potential applications in fields such as organic photovoltaics, sensors, and electronic devices. UV–Visible (UV-Vis) spectroscopy was employed to assess the optical properties of A2[Ru(bpy)_2_]^2+^, and it was observed to exhibit well-defined absorption bands typical of Ru(II) polypyridyl complexes. Additionally, the calculated optical band gap of A2[Ru(bpy)_2_]^2+^ was found to be 1.98 eV, a value consistent with similar structures in the literature and indicative of its potential for efficient light absorption and charge separation in various optoelectronic applications. Cyclic voltammetry (CV) measurements provided further insight into the electrochemical behavior of Ru(bpy)_2_]^2+^ revealing key redox processes associated with the Ru(II/III) and bipyridine ligand. These data not only reinforce the stability of the complex but also suggest its suitability for electron transfer applications in hybrid materials. Additionally, transient absorption experiments were conducted, providing complementary data on the excited-state dynamics and kinetics of electron transfer in the conjugated structure. In an effort to enhance the properties of Ru(bpy)_2_]^2+^ for potential hybrid applications, a 1,3-dipolar cycloaddition reaction was performed between sarcosine, reduced graphene oxide (RGO), and A2[Ru(bpy)_2_]^2+^. This reaction resulted in the formation of a hybrid material, RGO-A2[Ru(bpy)_2_]^2+^, combining the electron-rich properties of the conjugated oligomer with the high surface area and conductivity of graphene. This hybrid material is expected to exhibit improved electronic properties, potentially useful for applications in energy storage, catalysis, and flexible electronics. To confirm the successful covalent attachment of A2[Ru(bpy)_2_]^2+^ to RGO, several characterization techniques were employed. Raman spectroscopy showed a characteristic decrease in the ID/IG ratio, suggesting the functionalization of RGO with the A2[Ru(bpy)_2_]^2+^ complex. Scanning electron microscopy (SEM) revealed significant morphological changes in the RGO-A2[Ru(bpy)_2_]^2+^ hybrid compared to pure RGO, with the formation of rough surface textures indicative of the polymer’s presence. X-ray photoelectron spectroscopy (XPS) further confirmed the covalent attachment of the Ru complex, providing evidence of elemental ruthenium and nitrogen on the surface of the material, with the Ru 3d and N 1s peaks matching those expected for the A2[Ru(bpy)_2_]^2+^ complex. These combined spectroscopic, electrochemical, and morphological analyses provide strong indirect evidence of the successful synthesis and covalent integration of A2[Ru(bpy)_2_]^2+^ onto RGO, forming a hybrid material with promising properties for future applications in electronic and energy-related devices.

## Figures and Tables

**Table 1:** Summary of electro-optical results for A1[Ru(bpy)_2_]^2+^.

	E_1/2_(ox) (V)	E_1/2_(red) (V)	HOMO (eV)	LUMO (eV)	HOMO–LUMO Bandgap (eV)	Optical Bandgap (eV)
A1[Ru(bpy)_2_]^2+^	0.9 0.6	−1.0 −1.0	−5.7 −5.4	−3.8 −3.8	1.9 1.6	2.21
Literature structure (8b)	1.02	−1.32	−5.82	−3.48	2.34	2.12
A2[Ru(bpy)_2_]^2+^	0.6		−5.4			1.98
Literature structure (8b)	1.02	−1.32	−5.82	−3.48	2.34	2.12

**Table 2:** Resultant bandgaps of [A1[Ru]bpy^2+^]_2_Cl^−^.

[A1[Ru]bpy^2+^]2Cl^−^	E_g_ (eV)
Monomer	2.282
Dimer	2.232
Trimer	2.186

## Data Availability

Data will be available on request from authors.

## Supplementary Materials

The following supporting information can be downloaded at: https://www.mdpi.com/article/10.3390/ijms252312989/s1.
