# Supplementary material for: The Synthesis, Characterization, and Theoretical Study of Ruthenium (II) Polypyridyl Oligomer Hybrid Structures with Reduced Graphene Oxide for Enhanced Optoelectronic Applications
Source: Int J Mol Sci. 2024 Dec 3;25(23):12989. doi: 10.3390/ijms252312989 (PMC11640891; doi:10.3390/ijms252312989)
Supplement: Supplementary file 1 [file ijms-25-12989-s001.zip › ijms-3329679-supplementary.pdf]

Supporting Information

The Synthesis, Characterization, and Theoretical Study of Ruthenium (II) Polypyridyl Oligomer Hybrid Structures with Reduced Graphene Oxide for Enhanced Optoelectronic Applications

Table S1 Shows Total Energy, Band gap, and Dihedral angle of Carbon #24, #23, #18, and #19 in each geometry conformation

**FHIAIMS**	***Total (eV)***	***Bandgap (eV)***	***Dihedral angle***
**HF**	-57285.21313	8.339075	-52.2 º
**PBE/PBE0**	-57607.26535	3.270012	-44.8 º

Table S2 Shows Total Energy, Band gap, and Bond lengths between the Ruthenium ion and specified Nitrogen atoms for respective geometries. *Coordinates achieved from similar structures in the literature, relaxed via B3LYP settings[3]

**C989Ru chromophore**					
**FHI-aims**	*Total (eV)*	*Bandgap (eV)*	*Bond lengths*		
**HF**	No convergence	No convergence	No convergence		
**PBE/PBE0**	-215215.7302	2.603223	2.04 Å	Ru13 #13	N22 #22
			2.04 Å	Ru13 #13	N17 #17
			2.03 Å	Ru13 #13	N4 #4
			2.05 Å	Ru13 #13	N9 #9
**[Ru(bpy)2(bpy(O-)2)] ***			2.07 Å	Ru1 #1	N1 #1
			2.07 Å	Ru1 #1	N2 #2
			2.05 Å	Ru1 #1	N3 #3
			2.04 Å	Ru1 #1	N4 #4
			2.06 Å	Ru1 #1	N5 #5
			2.05 Å	Ru1 #1	N6 #6

Table S3 UV-Vis and cyclic voltammetry data taken of A1[Ru] Polymer

	$E_{1/2}$(ox) (V)	$E_{1/2}$(red) (V)	HOMO (eV)	LUMO (eV)	HOMO-LUMO bandgap (eV)	Optical bandgap (eV)
A1[Ru(bpy)$_2$]$^{2+}$	0.9<br>0.6	-1.0<br>-1.0	-5.7<br>-5.4	-3.8<br>-3.8	1.9<br>1.6	2.21
Literature structure (8b)	1.02	-1.32	-5.82	-3.48	2.34	2.12

Table S4 Confirmed energies, bandgaps, and bond lengths around the Ru^{2+} ion in A1[Ru]bpy

FHIAIMS	*Total (eV)*	*Bandgap (eV)*	*Bond lengths*		
**HF**	No convergence	No convergence	No convergence		
**PBE/PBE0**	-247688.9834	1.788993	2.07 Å	Ru149 #149	N4 #4
			2.08 Å	Ru149 #149	N9 #9
			2.07 Å	Ru149 #149	N16 #16
			2.08 Å	Ru149 #149	N21 #21
			2.08 Å	Ru149 #149	N161 #161
			2.08 Å	Ru149 #149	N172 #172

Table S5 FHI-aims simulation conclusions with Van der Waals considerations.

**FHI-aims Calculations with VDW considerations**					
**A1**	*Total Energy (eV)*	*Bandgap (eV)*	*Dihedral angle*		
**HF**	-57285.21313	8.339106	-55.2°		
**PBE0**	-57607.20198	3.266224	-44.1°		
**C989Ru**	*Total Energy (eV)*	*Bandgap (eV)*	*Bond Lengths*		
**HF**	No convergence	No convergence	No convergence		
**PBEPBE0**	-215215.7326	2.586318	2.03 Å	Ru13 #13	N4 #4
			2.04 Å	Ru13 #13	N9 #9
			2.03 Å	Ru13 #13	N17 #17
			2.04 Å	Ru13 #13	N22 #22
**A1[Ru] mono**	*Total Energy (eV)*	*Bandgap (eV)*	*Bond Lengths*		
**PBE0**	-247688.9403	1.80296244	2.06 Å	Ru149 #149	N4 #4
			2.07 Å	Ru149 #149	N9 #9

	2.06 Å	Ru149 #149	N16 #16
	2.07 Å	Ru149 #149	N21 #21
	2.08 Å	Ru149 #149	N161 #161
	2.07 Å	Ru149 #149	N172 #172

Table S6 Differences between FHI-aims calculations with and without Van der Waals force considerations

Total Energy (eV)			
**A1**	*w/o vdw*	*w/ vdw*	*Difference*
**HF**	-57285.21313	-57285.21313	1.494E-06
**PBEPBE0**	-57607.26535	-57607.20198	-0.063376
**C989Ru**	*w/o vdw*	*w/ vdw*	*Difference*
**HF**	No convergence	No convergence	No convergence
**PBEPBE0**	-215215.7302	-215215.7326	0.002398
**A1[Ru] mono**	*w/o vdw*	*w/ vdw*	*Difference*
**PBEPBE0**	-247688.9834	-247688.9403	-0.043100
**HOMO- LUMO Bandgap (eV)**			
**A1**	*w/o vdw*	*w/ vdw*	*Difference*
**HF**	8.339075	8.339106	-3.104E-05
**PBEPBE0**	3.270012	3.266224	0.003787
**C989Ru**	*w/o vdw*	*w/ vdw*	*Difference*
**HF**	No convergence	No convergence	n/a
**PBEPBE0**	2.603223	2.586318	0.01690434
**A1[Ru] mono**	*w/o vdw*	*w/ vdw*	*Difference*
**PBEPBE0**	1.788993	1.802962	-0.013968
